# JANUS, a spliceosome-associated protein, promotes miRNA biogenesis in Arabidopsis

**DOI:** 10.1093/nar/gkad1105

**Published:** 2023-11-22

**Authors:** Mu Li, Huihui Yu, Bangjun Zhou, Lu Gan, Shengjun Li, Chi Zhang, Bin Yu

**Affiliations:** Center for Plant Science Innovation, University of Nebraska-Lincoln, Lincoln, NE 68588–0666, USA; School of Biological Sciences, University of Nebraska-Lincoln, Lincoln, NE 68588–0118, USA; Center for Plant Science Innovation, University of Nebraska-Lincoln, Lincoln, NE 68588–0666, USA; School of Biological Sciences, University of Nebraska-Lincoln, Lincoln, NE 68588–0118, USA; Center for Plant Science Innovation, University of Nebraska-Lincoln, Lincoln, NE 68588–0666, USA; School of Biological Sciences, University of Nebraska-Lincoln, Lincoln, NE 68588–0118, USA; Center for Plant Science Innovation, University of Nebraska-Lincoln, Lincoln, NE 68588–0666, USA; School of Biological Sciences, University of Nebraska-Lincoln, Lincoln, NE 68588–0118, USA; Key Laboratory of Biofuels, Shandong Provincial Key Laboratory of Energy Genetics, Shandong Energy Institute, Qingdao New Energy Shangdong Laboratory, Qingdao Institute of Bioenergy and Bioprocess Technology, Chinese Academy of Sciences, Qingdao 266101, China; Center for Plant Science Innovation, University of Nebraska-Lincoln, Lincoln, NE 68588–0666, USA; School of Biological Sciences, University of Nebraska-Lincoln, Lincoln, NE 68588–0118, USA; Center for Plant Science Innovation, University of Nebraska-Lincoln, Lincoln, NE 68588–0666, USA; School of Biological Sciences, University of Nebraska-Lincoln, Lincoln, NE 68588–0118, USA

## Abstract

MicroRNAs (miRNAs) are important regulators of genes expression. Their levels are precisely controlled through modulating the activity of the microprocesser complex (MC). Here, we report that JANUS, a homology of the conserved U2 snRNP assembly factor in yeast and human, is required for miRNA accumulation. JANUS associates with MC components Dicer-like 1 (DCL1) and SERRATE (SE) and directly binds the stem-loop of pri-miRNAs. In a hypomorphic *janus* mutant, the activity of DCL1, the numbers of MC, and the interaction of primary miRNA transcript (pri-miRNAs) with MC are reduced. These data suggest that JANUS promotes the assembly and activity of MC through its interaction with MC and/or pri-miRNAs. In addition, JANUS modulates the transcription of some pri-miRNAs as it binds the promoter of pri-miRNAs and facilitates Pol II occupancy of at their promoters. Moreover, global splicing defects are detected in *janus*. Taken together, our study reveals a novel role of a conserved splicing factor in miRNA biogenesis.

## Graphical Abstract

**Graphical Abstract:**
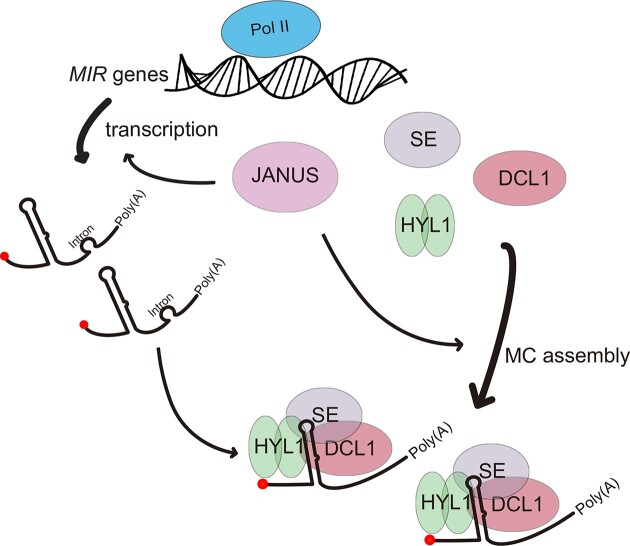

## Introduction

MicroRNAs (miRNAs) are 20–24 nucleotide (nt) non-coding RNAs that regulate gene expression through directing messenger RNA (mRNA) degradation and/or translational repression in both plants and animals (1–4). They have been implicated in various biological processes such as proliferation, differentiation, development, and stress responses (5–8). Most miRNAs are generated from primary miRNA transcripts encoded by the DNA-dependent RNA polymerase II (Pol II) dependent genes (*MIRs*) (9). In plants, the RNase III enzyme DICER-LIKE 1 (DCL1) forms a complex with SERRATE (SE; a C2H2 zinc finger protein), HYPONASTIC LEAVES 1 (HYL1; a double-stranded RNA-binding protein) and TOUGH (TGH, an RNA-binding protein) to co-transcriptionally process pri-miRNAs in the nucleus (10–14). After processing, miRNAs are 2′-O-methylated by the RNA methyltransferase HUA ENHANCER 1 (HEN1), loaded into the effector protein AGONAUTE 1 (AGO1) in the nucleus and exported to regulate target gene expression (15–21).

Multiple protein factors are dynamically associated with the DCL1 complex to regulate its activity and/or assembly. Notably, some these protein factors also contribute to pri-miRNA transcription or stability. Based on their functions, these protein factors can be divided into three groups. CBP80/20 (22–24), MOS2 (25), PNP1 (26), SICKLE (27), STA1 (28), TGH (12), THO1 (29) and THO2 (30) belong to one group, which contribute to efficient pri-miRNA process without effecting pri-miRNA transcription or stability. The second group includes CDC5(31), NOT2 (32), Elongator (33), MAC7 (34), PP4 (35), SMA1 (36), STV1 (37), THP1 (20) and CHR2 (38). These proteins modulate both pri-miRNA transcription and processing, suggesting that pri-miRNA transcription and processing are coordinately regulated. The third group contains HYL1 (39), SE (10), SEAP1 (40), DDL (41), MAC3 (42), PRL1 (43), MAC5 (44) and RACK1 (45). This group proteins play important roles in both pri-miRNA processing and stability, indicating that miRNA biogenesis is interconnected with general RNA metabolism. Moreover, regulation of miRNA biogenesis also occurs at post-translational levels. For instance, HYL1 activity and stability are modulated by phosphorylation and dephosphorylation (46–50) while SE phosphorylation triggers its degradation by 20S proteosome (51,52).

Splicing Factor 3B 4 (SF3B4)/Splicesome-associated protein 49 (SAP49) from mammals and its yeast counterpart Human Sap Homolog 49 (HSH49) are conserved spliceosome-associated proteins (53–55). They bind U2 small nuclear RNA (U2 snRNA) and are required for U2-snRNP assembly (56,57). SF3B4 also play important roles in transcription and translation of some genes in human (58–60). The Arabidopsis SF3B4 homolog named as JANUS has been shown to control pattern formation during early embryogenesis through modulating the transcription of WOX2 and PIN7 (61). However, its function in RNA metabolism remains largely unknown.

Here we report that JANUS plays important roles in miRNA biogenesis. JANUS interacts with DCL1 and SE, and directly binds the stem-loop of pri-miRNAs. The hypomorphic *janus* mutation reduces the activity of the DCL1 complex, causes reduced accumulation of miRNAs, and impairs the interaction of HYL1 with pri-miRNAs and the localization of HYL1 in the D-body, suggesting that JANUS may facilitate miRNA biogenesis by promoting the assembly of the DCL1 complex and the loading of pri-miRNAs into the DCL1 complex. Moreover, JANUS facilitate pri-miRNA transcription since the occupancy of Pol II at *MIR* promoter and *MIR* promoter activity are reduced in *janus*. Based on these observations, we propose that JANUS is a DCL1-associated protein that coordinates pri-miRNA transcription and processing.

## Materials and methods

### Plant materials and growth condition

The CS16053 (*janus-1*) line is in the Columbia (Col-0) background and was obtained from the Arabidopsis Biological Resources Center (ABRC). All plants were grown at 22°C with 16-h light and 8-h dark cycles. The primers used for genotyping are listed in Supplementary Table S1.

### Plasmids construction

*JANUS* cDNA was amplified by RT-PCR, cloned into pENTR/SD/D-TOPO, and subsequently cloned into pEarlyGate203 to generate the *p35S::MYC-SEAP1* plasmids, respectively. The artificial miRNA was designed using WMD3 (http://wmd3.weigelworld.org/cgi-bin/webapp.cgi). A synthetic fragment containing the artificial miRNA sequence and *MIR164A* backbone was cloned into pENTR/SD/D-TOPO, and subsequently cloned into pMDC32. The construct for CRISPR/Cas9-induced *JANUS* mutation was generated with binary vector pHEE401 using BsaI (NEB) and T4 ligase (NEB) according to the method described previously (62). To construct cCFP-JANUS, JANUS cDNA was PCR amplified and cloned into pSAT4-cCFP-C vector. Then, the *pro35S::cCFP-JANUS* fragment was released by I-SceI restriction enzyme digestion and subcloned to pPZP-RCS2-ocs-bar-RI vector. The construction of nVenus-DCL1, nVenus-HYL1, nVenus-SE, nVenus-CDC5, and nVenus-AGO1 was described previously (12). A 4.5 kb genomic fragment containing the *JANUS* promoter and coding regions was PCR amplified from wild-type plants, inserted into pENTR/SD/D-TOPO (K242020, Thermo Fisher Scientific), and subsequently cloned into the binary vector pEarlyGate 303 (63) to generate *pJANUS::JANUS-MYC*. The primers are listed in Supplementary Table S1.

### Complementation assay

The *pJANUS::JANUS-MYC* plasmid was transformed into *janus-1* and *janus-2*,and transgenic plants were identified through screening for Basta resistance.

### Gus histochemical assay

To visualize GUS expression, samples were immersed in the GUS staining solution (1 mM 5-bromo-4-chloro-3-indoly-β-d-glucuronic acid, 100 mM NaPO_4_ buffer, 3 mM each K_3_Fe(CN)_6_/K_4_Fe(CN)_6_, 10 mM EDTA and 0.1% NP-40) at 37°C for 5 h in the dark as described (42). The stained samples were treated with 70% ethanol to remove chlorophyll before imaging. Leaf surfaces were visualized with standard scanning electron microscopy preparative techniques.

### RNA extraction, RT-PCR, and quantitative RT-PCR

Total RNAs from inflorescences were extracted with TRI reagent (Molecular Research Center). To perform RT-PCR, total RNAs were treated with DNase I (Thermo Fisher Scientific) followed by reverse transcription using M-MLV reverse transcriptase (Promega) with oligo-d(T) primers according to manufacturer's instructions. RT-qPCR was performed using iCycler apparatus (Bio-Rad) with Accuris Instruments PR2000-N-100 Qmax Green Real Time PCR Mix. The primers are listed in Supplementary Table S1.

### Small RNA sequencing

Small RNA libraries were prepared using total RNAs extracted from inflorescences. Two biological replicates were performed. After sequencing, miRNAs are analyzed as described (12). After removing reads aligned to t/r/sn/snoRNA, the total numbers of perfectly aligned reads were used for normalization (Nobuta *et al.*, 2010).miRNA abundance was compared by using EdgeR with trimmed mean of M values normalization method (64). The dataset was deposited into National Center for Biotechnology Information Gene Expression Omnibus (Col-0 accession numbers: GSM7164784 and GSM7164785; *janus* accession numbers: GSM7164786 and GSM7164787).

### Differential gene expression and differential splicing analyses

RNA libraries were prepared using total RNAs extracted from inflorescences following standard protocol. Two biological replicates were performed. After sequencing, mRNAs are are analyzed as described (4). Differential gene expression analysis was conducted using R package Ballgown (58). Stattest function in Ballgown was used to test differential gene expression between Col-0 and *janus*. Only the genes with standard deviation of expression larger than 1 among all samples were used in the analysis and the genes with statistic *Q*-value ≤0.05 were considered as differentially expressed genes. Differential mRNA splicing analysis was conducted using R package VaSP (59). Only the introns supported with at least 5 junction reads in at least 1 sample were considered, and only the genes with the average read coverage larger than 1 in all samples were used in the analysis. Student's t-test was employed to test the difference of 3S scores between Col-0 and *janus*. Genes with any intron at P-value ≤ 0.05 and fold change of 3S scores ≥2 were considered as differential splicing genes. The dataset was deposited into National Center for Biotechnology Information Gene Expression Omnibus (Col-0 accession numbers: GSM7164780 and GSM7164781; janus accession numbers: GSM7164782 and GSM7164783).

### BiFC assay

The constructs of cCFP-JANUS, with nVenus-DCL1, nVenus-HYL1, nVenus-SE, nVenus-CDC5 and nVenus-AGO1 were co-infiltrated in *Nicotiana benthamiana* (*N*. *benthamiana*) leaves. After 48 hours, YFP and chlorophyll autofluorescence signals were observed by a confocal microscopy (Nikon A1 HD25).

### Co-IP assay

To examine the interaction of JANUS with DCL1 and SE, JANUS-FLAG was transiently co-expressed with GFP-DCL1 or MYC-SE in *N. benthamiana* as described (12). The expression of these transgenes was directed by the 35S promoter. Total proteins of infiltrated leaves were extracted with an extraction buffer (50 mM Tris–HCl 8.0, 150 mM NaCl, 5% glycerol, 5% Triton X-100, 1 mM EDTA, 1× complete protease inhibitor cocktail and 1 mM phenylmethylsulfonyl fluoride). Immunoprecipitation (IP) was performed on protein extracts using anti-GFP (GTA-20, Chromotek) or anti-FLAG antibodies (A4596, Sigma) coupled to protein G agarose beads. After IP, proteins were separated on a 10% SDS-PAGE and detected with western blot using monoclonal antibodies against GFP (902602, Biolegend) or FLAG (A8592, Sigma). DCL1, SE and HYL1 proteins in Arabidopsis were detected with antibodies against DCL1 (Agrisera, AS122102), SE (Agrisera, AS09532A) and HYL1 (Agrisera, AS06136).

### RIP analyses

RIP was performed as described (12). A total of ∼4 g inflorescences of Col or transgenic plants harboring a *p35S::MYC-JANUS* transgene were cross-linked with 1% formaldehyde for 10 min. Then, glycine was added to quench the reaction for 10 min. Following this step, nuclei were extracted and lysed in 400 uL nuclei lysis buffer (50 mM Tris–HCl pH 8.0, 10 mM EDTA, 1% SDS) by sonication five times. After debris was removed by centrifuge at 16000 × g for 10 min, equal amounts of proteins from various samples were diluted with RIP dilution buffer (16.7 mM Tris–HCl, 1.1% Triton X-100, 1.2 mM EDTA, pH 8.0, 167mM NaCl) and incubated with anti-MYC antibodies conjugated to protein G agarose beads or protein A/G agarose beads (for no-Ab controls). The immunoprecipitates were washed five times and then eluted with elution buffer (100 mM NaHCO3, 1% SDS) at 65°C. Following reversing cross-linking with proteinase K (Invitrogen) and 200 mM NaCl at 65°C, RNAs were extracted and used as templates for RT-PCR analyses. The primers are listed in Supplementary Table S1.

### ChIP assay

ChIP was performed using 14-day-old seedlings from Col-0 and *amiR^JANUS^* as described (Kim *et al.*, 2011). Anti-CTD antibody (ab817, Abcam) was used for immunoprecipitation. qPCR was performed on DNAs copurified with CTD antibody. The primers are listed in Supplementary Table S1.

## Results

### Identification of JANUS as a candidate acting in the miRNA biogenesis

We performed network analyses to identify candidate proteins involved in miRNA biogenesis in Arabidopsis using MAC5 as a bait, based on the fact that functionally related genes usually occur in the same gene network (65). The MAC5 network constructed with the STRING program (https://string-db.org) was consisted of known protein factors involved in miRNA biogenesis such as MAC3, PRL1, MAC7, CDC5 and others, and proteins acting in RNA metabolisms (Supplementary Figure S1A & B). From this network, we prioritized potential splicing-related proteins as our candidates since miRNA biogenesis is interconnected with RNA splicing processes. Among these candidates, we focused on JANUS (AT2G18510) because it plays essential roles in plant development (61) and (Supplementary Figure S2A–E), which is consistent with the role of miRNAs in controlling development.

**Figure 1.**
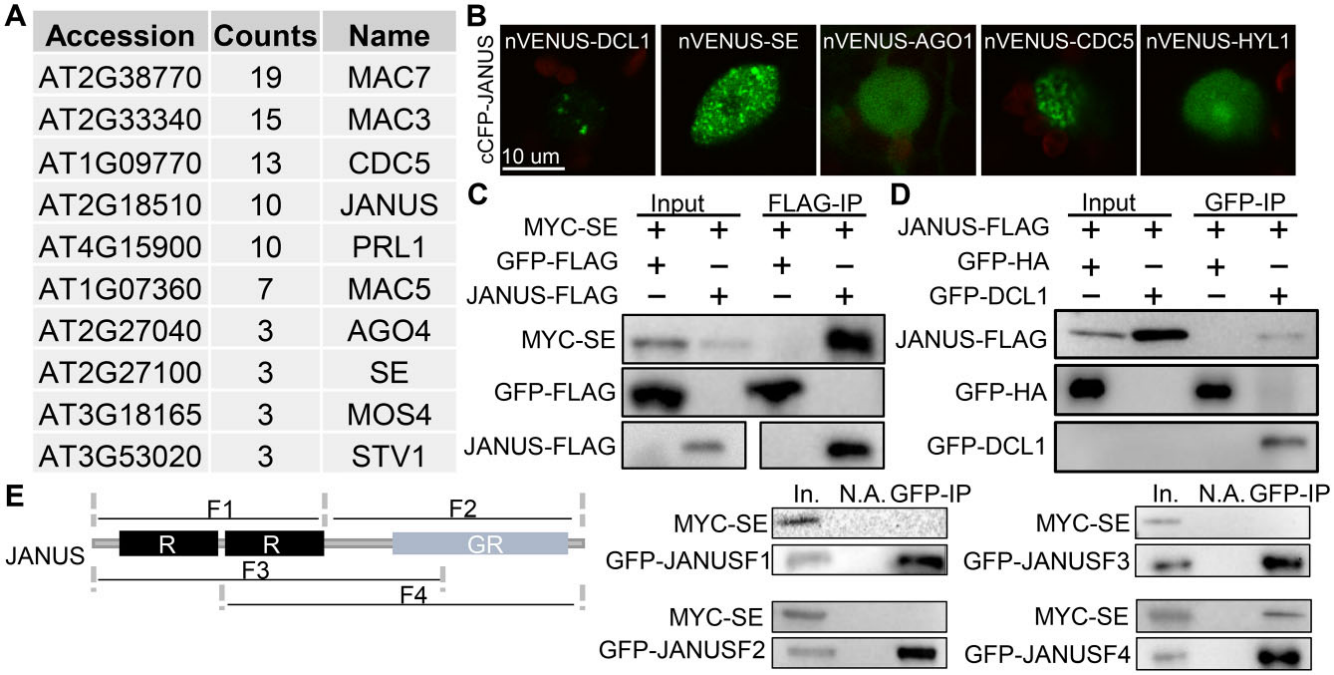
JANUS associates with DCL1 complex. **(A)** Protein factors identified from MYC-JANUS-associated proteins identified by mass spectrometry analysis. **(B)** Bimolecular fluorescence complementation (BiFC) analysis of JANUS with DCL1, HYL1, SE, AGO1 and CDC5. Paired cCFP- and nVENUS fusion proteins were co-expressed in *N. benthamiana*. Green color indicates the BiFC signal detected by a confocal microscopy at 48 h after infiltration. **(C)** Co-IP between JANUS-FLAG and MYC-SE. JANUS-FLAG or GFP-FLAG were co-expressed with MYC-SE in tobacco leaves. IPs were performed using anti-FLAG antibodies. JANUS-FLAG, FLAG and MYC-SE were detected by western blot. **(D)** Co-IP between JANUS-FLAG and GFP-DCL1. GFP-DCL1 or GFP-FLAG were co-expressed with JANUS-FLAG in *N. benthamiana*. IPs were performed using anti-GFP antibodies. JANUS-FLAG, GFP and GFP-DCL1 were detected by western blot. **(E)** Co-IP between truncated JANUS proteins and MYC-SE.JANUS protein contains two RNA recognition motifs (R) and a glycine-rich region (GR). Truncated JANUS proteins fused a GFP-tag at N-terminus. IPs were performed using anti-GFP antibodies. Truncated JANUS proteins and MYC-SE were detected by western blot.

In order to determine if JANUS could function in miRNA biogenesis, we analyzed JANUS interactome. We immunoprecipitated (IP) MYC-JANUS from transgenic plants harboring a *p35S::MYC-JANUS* transgene with anti-MYC antibodies, and performed mass spectrometry analyses. IP was also performed using the same anti-MYC antibodies with Col-0 as a negative control. JANUS pulled down several known protein factors involved in miRNA biogenesis including MAC3, MAC5, MAC7, CDC5, PRL1,STV1 and SE (Figure 1A), consistent with the result obtained from the functional network analysis of MAC5.

### JANUS is associated with the DCL1 complex

Interestingly, SE was also identified as an interactor of JANUS, suggesting that JANUS may associate with the DCL1 complex (Figure 1A). To test this possibility, we performed Bimolecular fluorescence complementation (BIFC) assay to test the potential interaction of JANUS with DCL1, HYL1, SE, CDC5 and AGO1. JANUS was fused with the C-terminal fragment of cyan fluorescent protein (cCFP), and DCL1, HYL1, SE, AGO1 and CDC5 were fused with the N-terminal fragment of Venus (nVenus). Co-expression of cCFP-JANUS with nVENUS-DCL1, -SE or -CDC5 produced strong YFP signals which were localized at the discrete bodies (Figure 1B), while co-expression of cCFP-JANUS with nVenus-HYL1 or - AGO1 pairs produced weak and diffused YFP signals. These results indicated that JANUS might associate with these proteins. Next, we performed co-IP assay to confirm the association of JANUS with the DCL1 complex. We transiently co-expressed JANUS-FLAG with either MYC-SE or GFP-DCL1 in *N*. *benthamiana* and performed IP with anti-FLAG or anti-GFP antibodies. After IP, SE was detected in the JANUS-FLAG precipitates and JANUS-FLAG was detected in the GFP-DCL1 precipitates (Figure 1C and D). Taken together, interactome and co-IP analysis show that JANUS interacts with DCL1 and associates with SE in Arabidopsis.

We further determined the protein domains of JNAUS that JANUS-SE interactions. JANUS harbors two RNA-recognition motifs (R1 and R2) at N-terminus and a glycine-rich motif (Pro-Gly motif; PG) at C-terminus (Figure 1E). We co-expressed four different JANUS fragments named F1 (aa 1–185, covering R1 and R2), F2 (aa 186–363; covering PG), F3 (aa 1–275; covering R1, R2 and a portion of PG), and F4 (aa 98–363; covering RRM2 and a portion of PG) with SE, respectively, in *N. benthamiana* (Figure 1E). F4, but not F1, F2 and F3 co-IPed with SE (Figure 1E), suggesting that RRM2 followed by PG is necessary and sufficient to interact with SE.

### JANUS is required for miRNA accumulation

**Figure 2.**
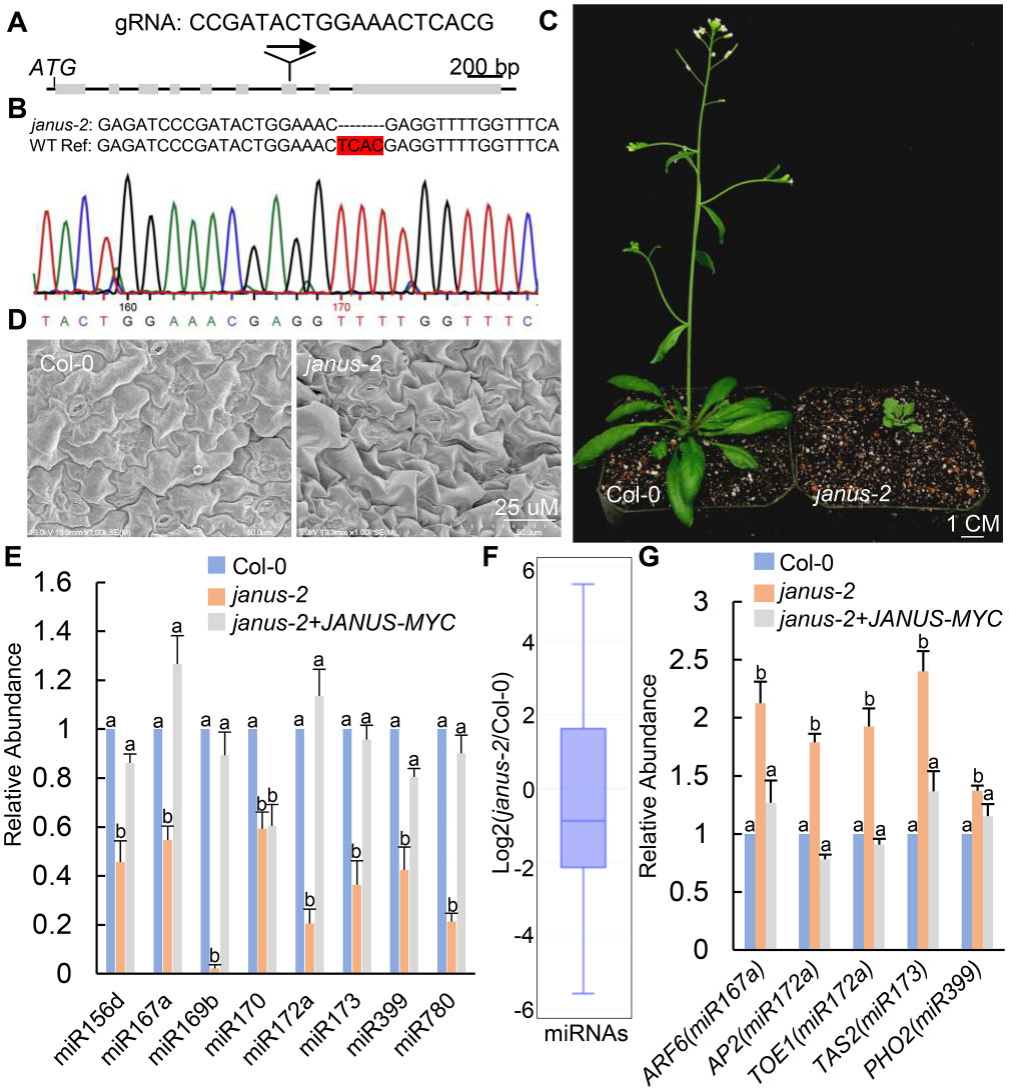
Hypomorphic *janus-2* mutation reduces miRNA accumulation. **(A)** SgRNA target site at *JANUS* gene. **(B)** Alignment of nucleotide sequences and sanger sequencing result at the target site in *janus-2*. The letters highlighted in red indicate four-bp deletion. **(C)** Five-week-old plants of Col-0 and *junus-2*. **(D)** Scanning electron microscopy of Col-0 and *junus-2* leaf surfaces. **(E)** The relative levels of miRNAs detected by RT-qPCR. miRNA levels in Col-0, *junus-2* and *junus-2* harboring the *pJANUS::JANUS-MYC* transgene. The levels of miRNAs were normalized to those of U6 RNAs and compared with Col-0 (set as 1). Different letters indicate significant difference determined by ANOVA (*P* < 0.05). **(F)** Small RNA sequencing analysis in Col-0 and *junus-2*. The miRNA abundance was calculated as reads per million, and a log2-transformed ratio of *junus-2*/Col-0 was plotted. **(G)** The levels of miRNA target transcripts in Col-0 *junus-2* and *junus-2* harboring the *pJANUS::JANUS-MYC* transgene detected by RT-qPCR. The levels of miRNA target transcripts were normalized to those of *UBQ5* and compared with Col-0 (set as 1). Different letters indicate significant difference determined by ANOVA (*P* < 0.05).

To evaluate if JANUS functions in miRNA biogenesis, we used a CRISPR/CAS9 system (62) to generate a weak allele of *JANUS*. In T1 generation, we obtained a hypomorphic *janus* mutant line, which contains a four-bp deletion in the seventh exon of JNAUS, and named it as *janus-2* (Figure 2A and B). The heterozygous *janus-2*/+ line was backcrossed to Col-0 for three times to remove the CRISPR/CAS9 transgene and other potential mutations. The resulting *janus-2* mutant displayed pleiotropic development defects such as smaller plant size, serrated leaves, and entirely abolished fertility (Figure 2C, Supplementary Figure S2D). Moreover, the pavement cells in *janus-2* were smaller than those in Col-0 as observed by scanning electronic microscope (SEM) (Figure 2D). Expression of a *pJANUS::JANUS-MYC* transgene fully recovered the developmental defects of *janus-2* (Supplementary Figure S2D and E). In addition, we found that down regulation of *JANUS* transcripts with an artificial miRNA (*amiR^JANUS^*) (Supplementary Figure S3A) also caused delayed growth of plants (Supplementary Figure S3B and C). These results demonstrated that JANUS is required for plant development.

We next tested miRNA accumulation in inflorescences of *janus*-2. Indeed, reverse transcription quantitative PCR (RT-qPCR) analyses showed that several examined miRNAs were reduced in *janus-2* relative to Col-0 (Figure 2E). The miRNA levels were restored in *janus-2* harboring the *pJANUS::JANUS-MYC* transgene (Figure 2E). In addition, miRNA levels were also reduced in abundance in *amiR^JANUS^* relative to Col-0 (Supplementary Figure S3D). Illumina deep sequencing further confirmed that the accumulation of miRNAs was globally reduced in *janus-2* relative to Col-0 (Figure 2F and Supplementary dataset 1). These results demonstrate that JANUS is required for miRNA accumulation.

We next performed reverse transcription quantitative PCR (qRT-qPCR) to examine the transcript levels of several miRNA target transcripts including *ARF6*, *AP2*, *TOE1*, *TAS2* and *PHO2*, which are targets of miR167, miR172, miR173 and miR399, respectively. The levels of these targets were moderately increased in *janus-2* compared with Col-0 and the complementation lines (Figure 2G). This result is consistent with the decreased levels of miRNAs in *janus-2*.

### JANUS regulates the transcription of *MIR*

**Figure 3.**
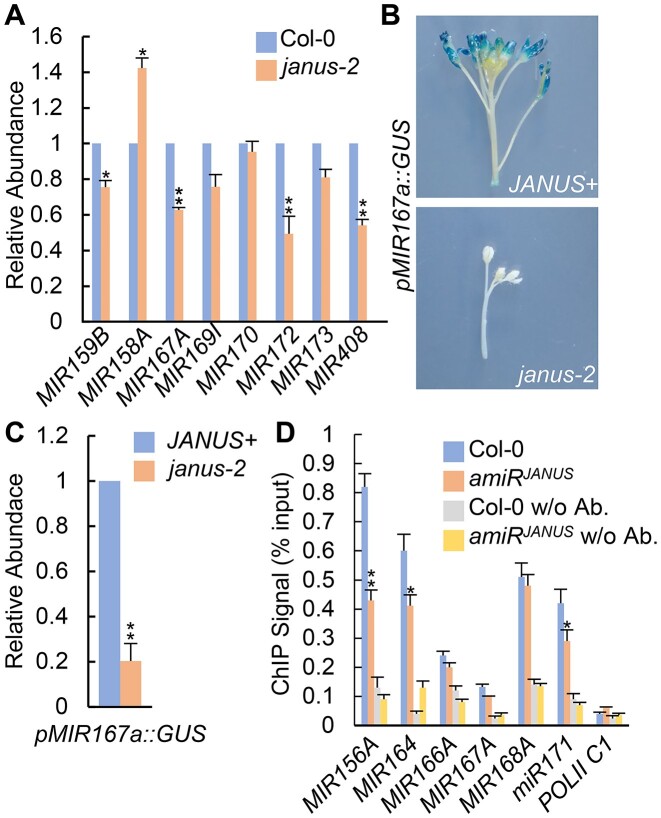
JANUS is required for the transcription of pri-miRNAs. **(A)** The accumulation of pri-miRNAs in Col-0 and *janus-2* detected by RT-qPCR. Pri-miRNA levels in *janus-2* were normalized to those of *UBQ5* and compared with Col-0 (set as 1). Error bars: standard deviations (SD) of three replicates. **P < 0.01, *P < 0.05 (Student's *t* test). **(B)** The levels of GUS in *JANUS +* and *janus-2* harboring *pMIR167a::GUS*. *JANUS+*: *JANUS/JANUS*, or *JANUS/janus-2*.**(C)** The transcript levels of *GUS* driven by *MIR167a* promoter in *JANUS +* and *janus-2*. GUS transcript levels were determined by qRT-PCR. The *GUS* mRNA levels in *janus-2* were normalized to UBQ5 and compared with those in *JANUS+*. *P < 0.05, **P < 0.01 (Student's *t* test). **(D)** The occupancy of Pol II at *MIR* promoters in Col-0 and *amiR^JANUS^* was detected by chromatin immunoprecipitation (ChIP) followed by qPCR. IP was performed using antibodies against CTD on protein extracts from Col-0 and *janus-2*. The intergenic region between At2g17470 and At2g17460 (POL II C1) was amplified as a negative control. *P < 0.05, **P < 0.01 (Student's t test).

Next we asked how JANUS acts in miRNA biogenesis. Because JANUS has been shown to regulate the transcription of several genes (66), we tested if *janus-2* affects the transcript levels of several genes involved in miRNA biogenesis including *CBP20*, *CBP80*, *DCL1*, *DDL*, *HEN1*, *HYL1* and *SE*, and pri-miRNA levels through RT-qPCR. While the transcript levels of miRNA biogenesis related genes did not show obvious change (Supplementary Figure S4), pri-miRNA levels were reduced in both *janus-2* and *amiR^JANUS^* relative to Col (Figure 3A and Supplementary Figure S3E), suggesting that JANUS may promote pri-miRNA transcription. We tested this possibility using a GUS reporter gene driven by the *MIR167a* promoter (*pMIR167a::GUS*), which has been used to examine the function of several transcription factors in modulating pri-miRNA transcription (31,36). We crossed *janus-2*/+ with the transgenic line harboring the *pMIR167a::GUS* transgene and identified *JANUS*+ (JANUS/ JANUS or JANUS/ *janus-2*) and *janus-2* harboring the *pMIR167a::GUS* transgene. GUS staining on these plants revealed that the GUS activity was lower in *janus-2* than that in *JANUS*+ (Figure 3B). qRT-PCR analysis confirmed that the GUS mRNA levels in *janus-2* were reduced significantly relative to those in *JANUS*+ (Figure 3C). Then we used chromatin immunoprecipitation (ChIP) assays to examine the occupancy of Pol II at five *MIR* promoters in Col-0 and *amiR^JANUS^* with anti-RPB2 antibody. qPCR analysis showed that the occupancy of Pol II at three *MIR* promoters were reduced in *amiR^JANUS^* relative to Col-0 (Figure 3D). Taken together, these results show that JANUS affects *MIR* transcription.

### JANUS interacts with Pri-miRNAs *in vitro* and *in vivo*

**Figure 4.**
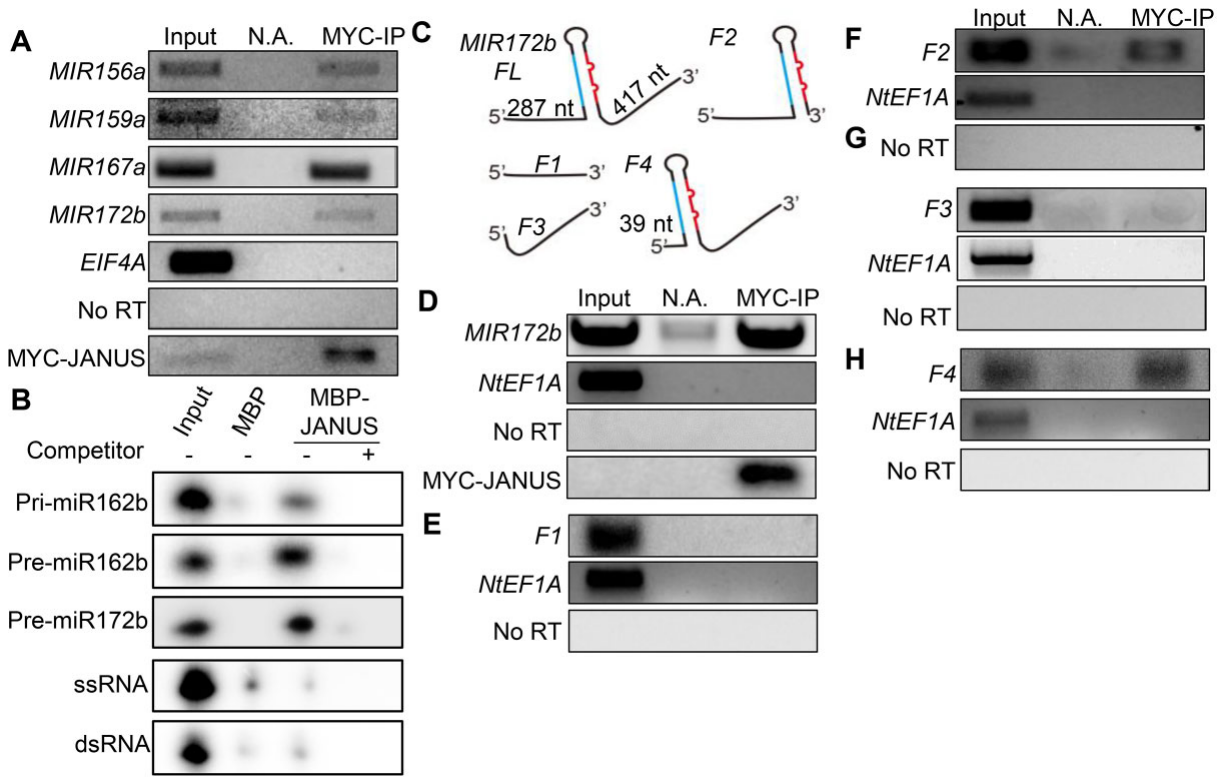
JANUS binds the stem-loop region of pri-miRNAs. **(A)** JANUS binds pri-miRNAs *in vivo*. RIP assay was performed on the transgenic plants harboring *p35S::MYC-JANUS* using anti-MYC antibodies. After RIP, RNA was extracted and detected by RT-PCR. No Ab means no antibody control. *EIF4A* was used as negative control. **(B)** JANUS binds pri-miR162b, pre-miR162b and pre-miR172b *in vitro*. ssRNA: single stranded-RNA; dsRNA: double stranded-RNA. **(C)** Diagrams of various *MIR172b* constructs used for the JANUS-binding assay. miR172 is shown in red; miR172b* is shown in blue. **(D–H)** The interaction of JANUS with full-length and truncated *MIR172b* RNAs. MYC-JANUS and *MIR172b* were transiently co-expressed in tobacco leaves. IP was performed with anti-MYC antibodies. *NtEF1A* was used as negative control. No Ab: IP without antibody.

Because JANUS is a putative RNA-binding protein, we investigated if JANUS binds pri-miRNAs *in vivo*. We performed an RNA immunoprecipitation assay (RIP) on the inflorescences of the transgenic plants harboring the *p35S::MYC-JANUS* transgene. Following cross-linking, nuclear isolation, and IP, RT-PCR was performed, and we can detect the enrichment of *pri-miR156a*, *pri-miR159a*, *pri-miR167a* and *pri-miR172b* in the MYC-JANUS IPs, but not in the no-antibody control (Figure 4A). Moreover, the negative control *EIF4A* was not detected in the MYC-JANUS IPs (Figure 4A). These results demonstrate that JANUS binds pri-miRNAs *in vivo*. Next, we performed an *in vitro* pull-down assay to examine whether JANUS could directly bind *pri-miR162b*. We expressed MBP and MBP-JANUS in *E. coli* and purified them with amylose resin (Supplementary Figure S5). Then we incubated MBP and MBP-JANUS with radioactive labeled *pri-miR162b*, which was transcribed *in vitro*. MBP-JANUS, but not MBP, was able to pull down pri-miR162b. MBP-JANUS can also bind the *precusor-miR162b* (pre-miR162b) and precusor-miR172b (pre-miRN172b) (Figure 4B). We also examined if JANUS binds dsRNAs and ssRNAs. However, we did not detect the interaction of JANUS with dsRNAs or ssRNAs (Figure 4B).

To examine which region(s) within pri-miRNAs can be recognized by JANUS, we used an *in vivo* assay developed from our previous studies (Figure 4C) (37,44). We first examined the interaction of MYC-JANUS with *pri-miR172b* in *N. benthamiana* transiently expressing *p35S::MYC-JANUS* and *p35S::MIR172b* which contains a full length *pri-miR172b* transcript (*MIR172bFL*). Consistent with our RIP assay, MYC-JANUS binds *MIR172bFL*, but not the endogenous control *NtEF1A* RNA from *N. benthamiana* (Figure 4D). We further investigated the interaction of MYC-JANUS with the *MIR172bF1* (a 287-nt 5′ arm), *MIR172bF2* (a 287-nt 5′arm + a stem loop region + a 6-nt 3′ arm), *MIR172bF3* (a 417-nt 3′ arm), *MIR172F4* (a 39-nt 5′ arm + a stem loop region + a 6-nt 3′ arm) (Figure 4D). MYC-JANUS was able to bind *MIR172bF2*, *MIR172bF4*, but not *MIR172bF1* and *MIR172bF3* (Figure 4E–H). These results together with the *in vitro* pull-down assay suggest that JANUS may bind the stem-loop region of pri-miRNAs and require imperfect base-pair or the junction between dsRNA and single-stranded in the stem-loop.

### JANUS is required for the formation of the D-Bodies

**Figure 5.**
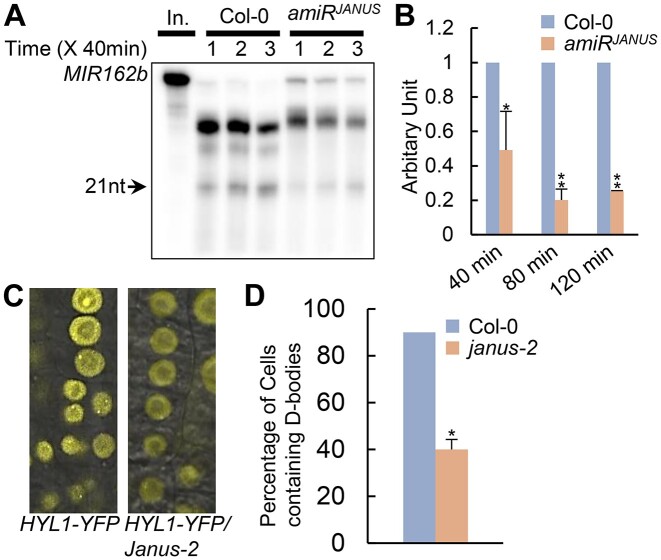
JANUS is required for the maturation of miRNA, the localization of HYL1 and the interactions between HYL1 and pri-miRNAs. **(A)** The amount of miR162b produced from *MIR162b* was reduced in *amiR^JANUS^*. Proteins were isolated from seedlings of Col-0 and *amiR^JANUS^* and incubated with *MIR162b*. The reactions were stopped at various time points as indicated in the picture. **(B)** Quantification of miR162b production in *amiR^JANUS^* compared to that in Col-0. The radioactive signal of miR162 were normalized to input and compared with that of Col-0. The amount of miR162 produced in Col-0 was set as 1. The value represents mean of two repeats. *P < 0.05, **P < 0.01 (Student's *t* test). **(C)** Image of HYL1 localization in the cells of root elongation region of Col-0 and *janus-2*. Ten-day-old plants were examined. **(D)** Quantification of root cells harboring HYL1-localized D-bodies in Col-0 and *janus-2*. Over 100 cells for each genotype were examined. Error bar indicates SD. **P < 0.01 (Student's *t* test).

The interaction of JANUS with DCL1 and SE led us to test if JANUS could modulate pri-miRNA processing using an *in vitro* assay (12). In this assay, we incubated radioactive labeled *pri-miR162b* with the protein extracts from young flower buds of Col, *or amiR^JANUS^*. The production of miR162 was reduced in the protein extracts of *amiR^JANUS^* relative to Col (Figure 5A and B), suggesting that JANUS may promote pri-miRNA processing. Next, we tested the effect of *janus-2* on the formation of the D-body using HYL1-YFP as a reporter gene (25). We crossed a HYL1-YFP transgenic line into *janus*-2/+ and calculated the percentage of cells containing D-bodies in the root tips and elongation region. As previously reported (34,42), the HYL1-containing D-bodies existed in most cells (∼90%; Figure 5C and D) in *JANUS*+ (JANUS/ JANUS or JANUS/ *janus-2*), whereas D-bodies were observed in only ∼40% of cells in *janus-2*. This result demonstrates that JANUS is required for correct HYL1 localization, revealing its potential role in facilitating D-body formation.

### The interaction of HYL1 with pri-miRNAs is impaired in *amiR^JANUS^*

SAP49 binds the pre-mRNA-U2 snRNA helix to promote the interaction between U2 snRNP and pre-mRNAs (56,57). By analogy, we sought that JANUS might also facilitate the interaction of pri-miRNA with the DCL1 complex. Therefore, we tested the interaction of HYL1 with pri-miRNAs using an RNA IP (RIP) assay, which is an indicator of pri-mRNA loading (12). HYL1 was IPed from protein extracts of *amiR^JANUS^* and Col-0 using antibodies recognizing HYL1 (Supplementary Figure S6), and qRT-PCR was performed to examine the amounts of pri-miRNAs associated with HYL1. The result showed that three examined HYL1-bound pri-miRNAs were reduced in abundance in *amiR^JANUS^* relative to Col-0 (Supplementary Figure S6). This result shows that JANUS may enhance pri-miRNA loading to the DCL1 complex.

### JANUS affects gene expression at global levels

**Figure 6.**
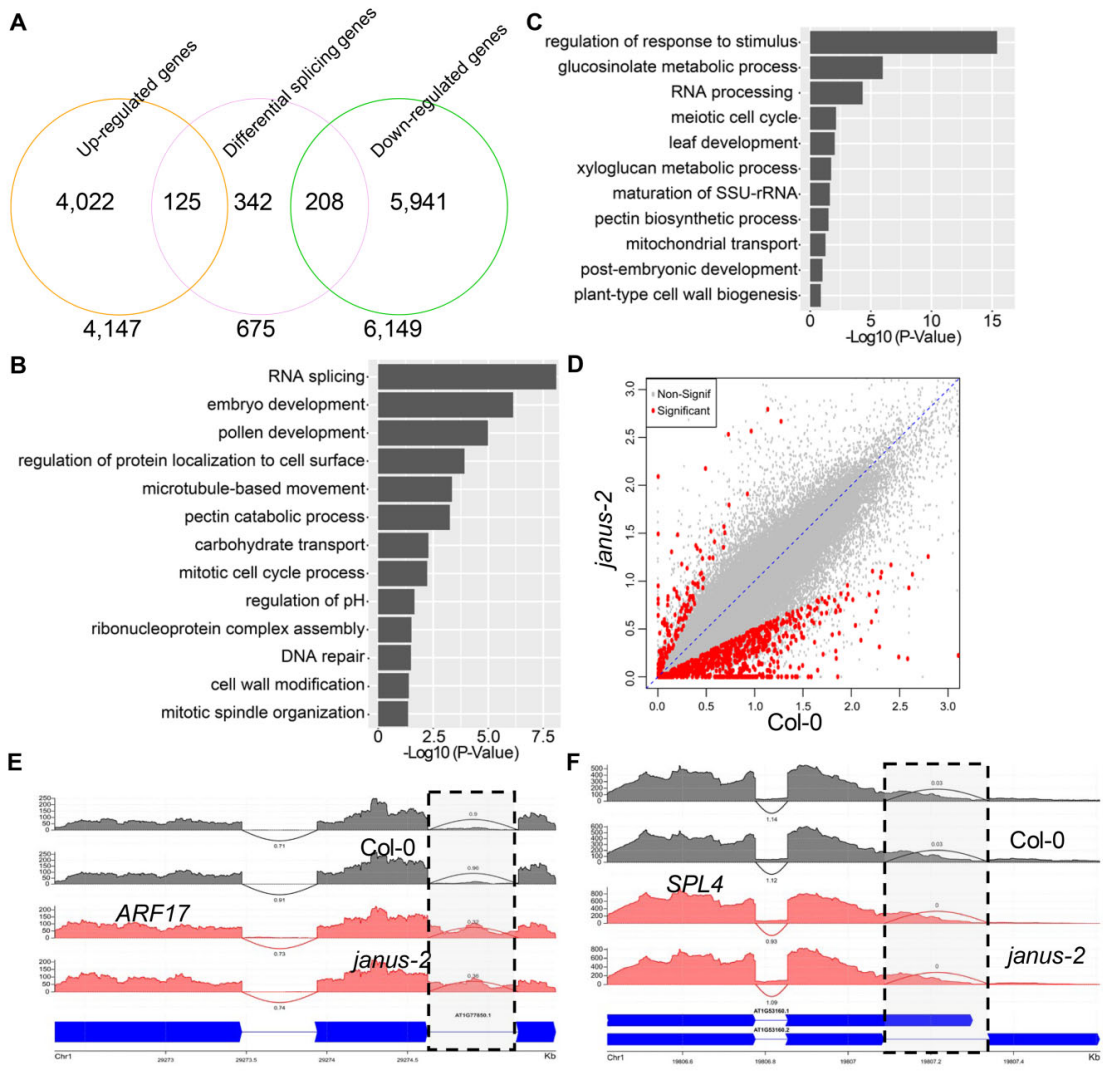
JANUS affects gene expression and splicing at global levels. **(A)** Venn diagram showing the degree of overlap of splicing defective genes with up-regulated or down-regulated in *janus-2* relative to Col-0. **(B)** GO enrichment of down-regulated genes in *janus-2* relative to Col-0. **(C)** GO enrichment of up-regulated genes in *janus-2* relative to Col-0. **(D)** Genome − wide intron Single Splicing Strength (3S) scores in Col-0 and *janus-2*. The red dots indicate introns with significant differential splicing between Col-0 and *janus-2*.and the gray dots indicate no significant difference. **(E** and **F)** Two examples of differentially spliced transcripts between Col-0 and *janus-2*. For each transcript, the x-axis is the genomic position, the y-axes are the RNA-seq read numbers, the arcs indicate exon-exon junctions (introns) and the numbers are Single Splicing Strength (3S, see Methods) scores. Transcripts from Col-0 are in grey. Transcripts from *janus-2* are in pink and gene structures are in blue. The significant differentially spliced introns are highlighted in vertical gray box.

Next we examined the general role of JANUS in modulating gene expression. We compared mRNA profiling in the flowers from the *janus-2* with that in WT by RNA-Seq analyses in two biological replicates. After sequencing, we identified DEGs between *janus-2* and Col-0 with fold change of 1.5 or more. A total of 6149 down-regulated and 4147 up-regulated genes were identified, respectively, in *janus-2* (Figure 6A and Supplementary dataset 2). To better understand the function of JANUS, we performed gene ontology (GO) analyses on DEGs in *janus-2*. Genes related to RNA splicing, embryo development, pollen development, ribonucleoprotein complex assembly, cell wall modification, DNA repair were enriched in down-regulated genes (Figure 6B and Supplementary dataset 3), while genes involved in regulation of response to stimulus, glucosinolate metabolism, RNA processing, meiotic cell cycle and leaf development were detected in up-regulated genes (Figure 6C and Supplementary dataset 3).

### JANUS promotes pre-mRNA splicing

Next, we asked if JANUS also plays a role in splicing by examining the effect of *janus-2* on intron retention of pre-mRNAs. The ratio between RNA-Seq reads mapped to introns and those mapped to exons was used to calculate the intron retention. The intron retention at global levels was determined using all annotated transcripts that passed the abundance filter. The result showed that the intron retention rate in *janus-2* was higher than in WT. A total of 675 genes were found to have higher intron retention rate in *janus-2* relative to WT (Figure 6D and Supplementary dataset 4). Figure 6E showed two examples of impaired mRNA intron retention in *janus-2* relative to WT. We randomly selected three genes with increased intron retention in *janus-2* for validation using RT-PCR analysis. The intron retention was increased in all these three genes in *janus-2*, agreeing with the RNA-Seq result (Supplementary Figure S7A and B). We next asked if splicing defects caused altered expression levels in *janus-2*. The co-occurrence of genes with intron retention defects with both upregulated and downregulated DEGs were examined. Only a small portion of DEGs had splicing defects (Figure 6A).

## Discussion

SF3B4/HSH49 is a conserved U2-snRNP assembly factor from yeast to human (53–55). Its role in regulating transcription and translation of specific genes have also been reported (58–60). However, the function of JANUS, a homolog gene of SF3B4/HSH49 from plant remains largely unknown. In this study, network analysis suggests that JANUS is functionally correlated with MAC5. Moreover, JANUS is physically associated with the MAC complex and the DCL1 complex, suggesting that it may function in miRNA biogenesis. Indeed, both the weak *janus-2* mutation and knockdown of *JANUS* by *amiR^JANUS^* cause pleiotropic development defects and reduce the accumulation of miRNA.

JANUS appear to affect pri-miRNA processing, based on the facts that miRNA levels are reduced in *janus* and that the production of miR162 from both pri-miR162b and pre-miR162b is reduced in *janus* protein extract relative to WT. How does JANUS promote pri-miRNA processing? It may promote DCL1 activity through its interaction with the DCL1 complex. In human and yeast, JANUS homologs are considered as a platform for the U2 snRNP assembly through their interactions with other proteins (67). We suspect that JNAUS may play a similar role in miRNA biogenesis. Supporting this notion, the number of D-bodies is reduced in *janus-2*. In addition, it may directly modulate DCL1 activity through its R2-PG domain. In human, SF3B4 modulates the activity of Bone morphogenetic protein (BMP)-2 through R2-PG-mediated SF3B4-BMP-2 interaction (68). We propose that JANUS may have a similar effect on SE. Interestingly, the R2 domain of JANUS also interact with Pol II (61), suggesting that JANUS may coordinate co-transcriptional processing of pri-miRNAs. JANUS may also promote pri-miRNA processing through its interaction with pri-miRNAs. The assembly of RNA into protein complex or efficient RNA processing often needs conformation changes. Our results show that JANUS binds the stem-loop of pri-miRNAs and promotes the interaction of HYL1 with pri-miRNAs. Thus, it is possible that JANUS may alter the structure of pri-miRNAs to facilitate their assembly into the DCL1 complex. Indeed, SF3B4/HSH49 binds the U2 snRNA and pre-mRNA to alter their structure, and thereby promotes their interaction with U2snRNP (69).

JANUS may also affect the accumulation of some miRNAs through its effect on pri-miRNAs. The levels of some pri-miRNAs are reduced in *janus*, which could be caused by reduced stability or transcription. The interaction of JANUS with Pol II together with the fact that Pol II occupancy at some *MIR* promoters and *MIR167a* promoter activity are reduced in *janus* demonstrate that JNAUS likely promotes the transcription of some pri-miRNAs. It has been shown that JANUS] promotes the transcription of WOX2 and PIN7 during early embryogenesis. These results suggest that like SF3B4/HSH49, JANUS modulates the transcription of a subset of genes including some pri-miRNAs. Indeed, our RNA seq-analysis suggest that the transcript levels of many genes are reduced in *janus*.

The association of JANUS with MAC suggest that like SF3B4/HSH49m, JANUS may play a role in splicing. Indeed, RNA-seq analysis shows that intron retention of many pre-mRNAs is altered in *janus*. Interestingly, only few pri-miRNAs have intron retention defects, indicating that JANUS may act in splicing independent of its function in miRNA biogenesis. Notably, the transcript levels of genes with altered intron retention can be up-regulated, down-regulated or unchanged, showing that the correlation between intron defection and changes in transcript levels is not significant. This result suggests that JANUS a broad role in RNA metabolism.

To summarize, our work uncovers a role of JANUS in miRNA biogenesis. It may promote miRNA accumulation through facilitating the assembly of the DCL1 complex and the interaction of pri-miRNAs with the DCL1 complex, and /or functioning as accessory protein factor to enhance DCL1 activity. Beside this function, JANUS also plays essential role in splicing and modulates the transcription of a subset of genes. Given the function of SF3B4/HSH49m in regulating translation of specific genes (53), it is possible JANUS has additional functions in plants. Through these combined functions, JANUS ensures the proper development of plants.

## Supplementary Material

gkad1105_Supplemental_Files

## Data Availability

For mRNA profiling, the dataset was deposited into National Center for Biotechnology Information Gene Expression Omnibus (Col-0 accession numbers: GSM7164780 and GSM7164781; janus accession numbers: GSM7164782 and GSM7164783). For small RNA profiling, the dataset was deposited into National Center for Biotechnology Information Gene Expression Omnibus (Col-0 accession numbers: GSM7164784 and GSM7164785; *janus* accession numbers: GSM7164786 and GSM7164787).
