# Supplementary material for: JANUS, a spliceosome-associated protein, promotes miRNA biogenesis in Arabidopsis
Source: Nucleic Acids Res. 2023 Nov 22;52(1):420–30. doi: 10.1093/nar/gkad1105 (PMC10783502; doi:10.1093/nar/gkad1105)
Supplement: gkad1105_Supplemental_Files [file gkad1105_supplemental_files.zip › Supplemental Materials.docx]

**
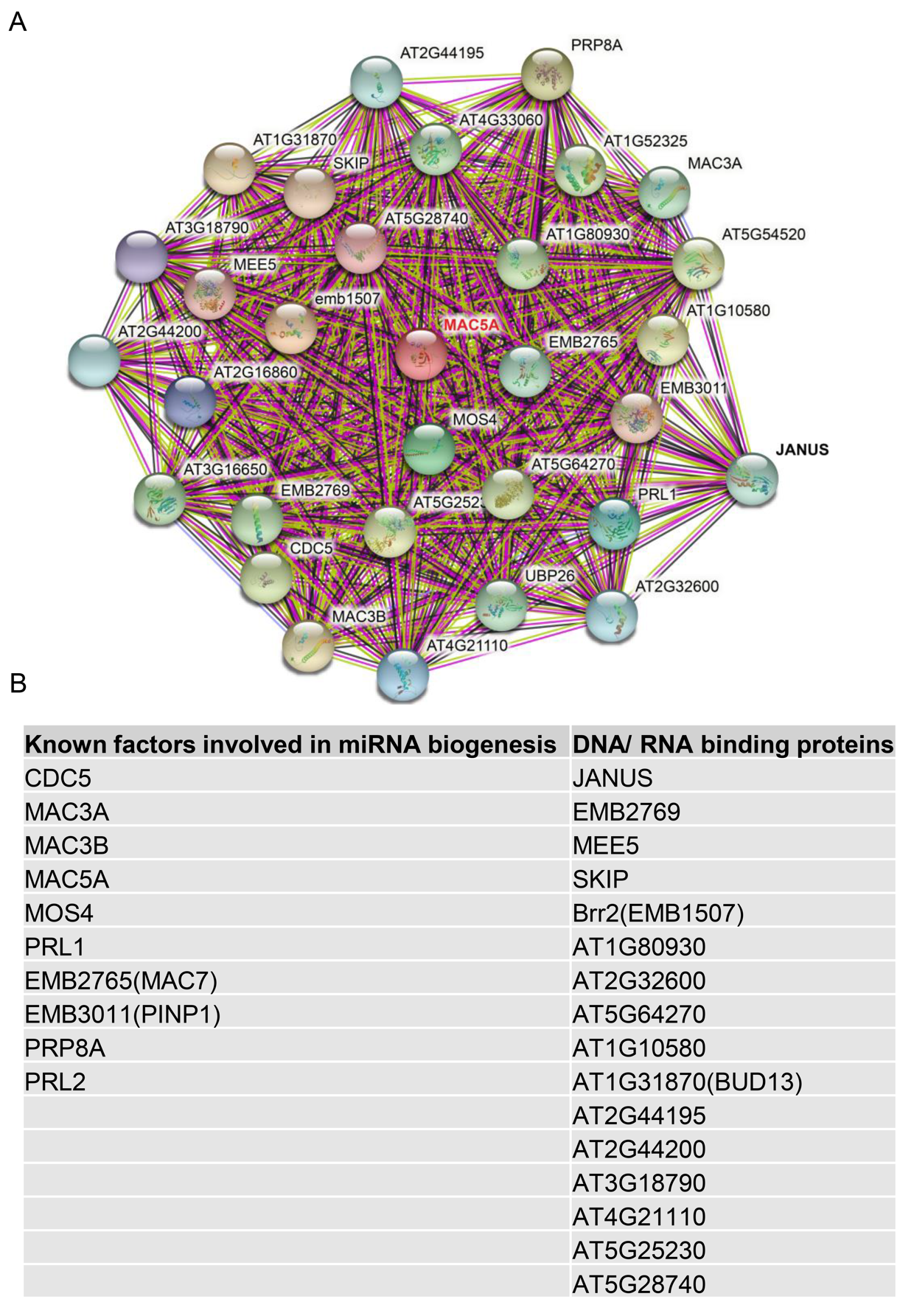
**

**Supplementary Figure S1. Functional gene network analysis of MAC5.** (A) Functional network of MAC5 was constructed using STRING program with a high confidence score of 0.7. Nodes and lines represent genes and functional links, respectively. Line color indicates the type of interaction evidence. Magenta: experimental data; light blue: association in curated databases; black: co-expression. (B) List of genes involved in miRNA biogenesis and putative DNA/RNA binding proteins in the MAC5 functional gene network.

**
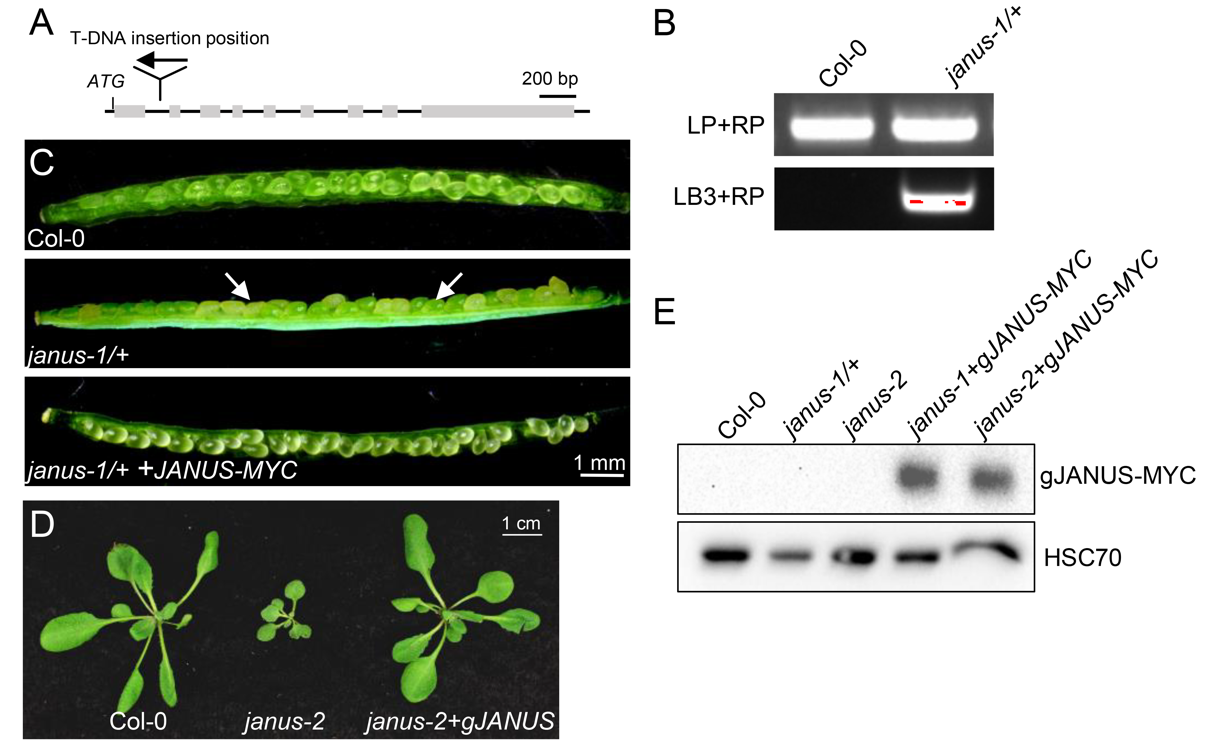
**

**Supplementary Figure S2. Analysis of *janus-1* and *janus-2*.** (A) The insertion site of *janus-1* is shown above the *JANUS* gene structure. (B) Detection of the T-DNA insertion in *janus-1/+* by PCR analysis. The LP/RP and LB3/RP primer pairs were used to identify JANUS and the T-DNA flanking sequences, respectively. (C) Siliques of Col-0, *janus-1* and transgenic plants harboring *pJANUS::JANUS-MYC*. (D) Three-week-old plants of Col-0, *janus-2* and transgenic plants harboring *pJANUS::JANUS-MYC*. (E) JANUS-MYC protein in the transgenic plants harboring *g*JANUS *1-MYC* detected by western blot. Heat Shock Protein 70 (HSC70) used as the loading control.

**
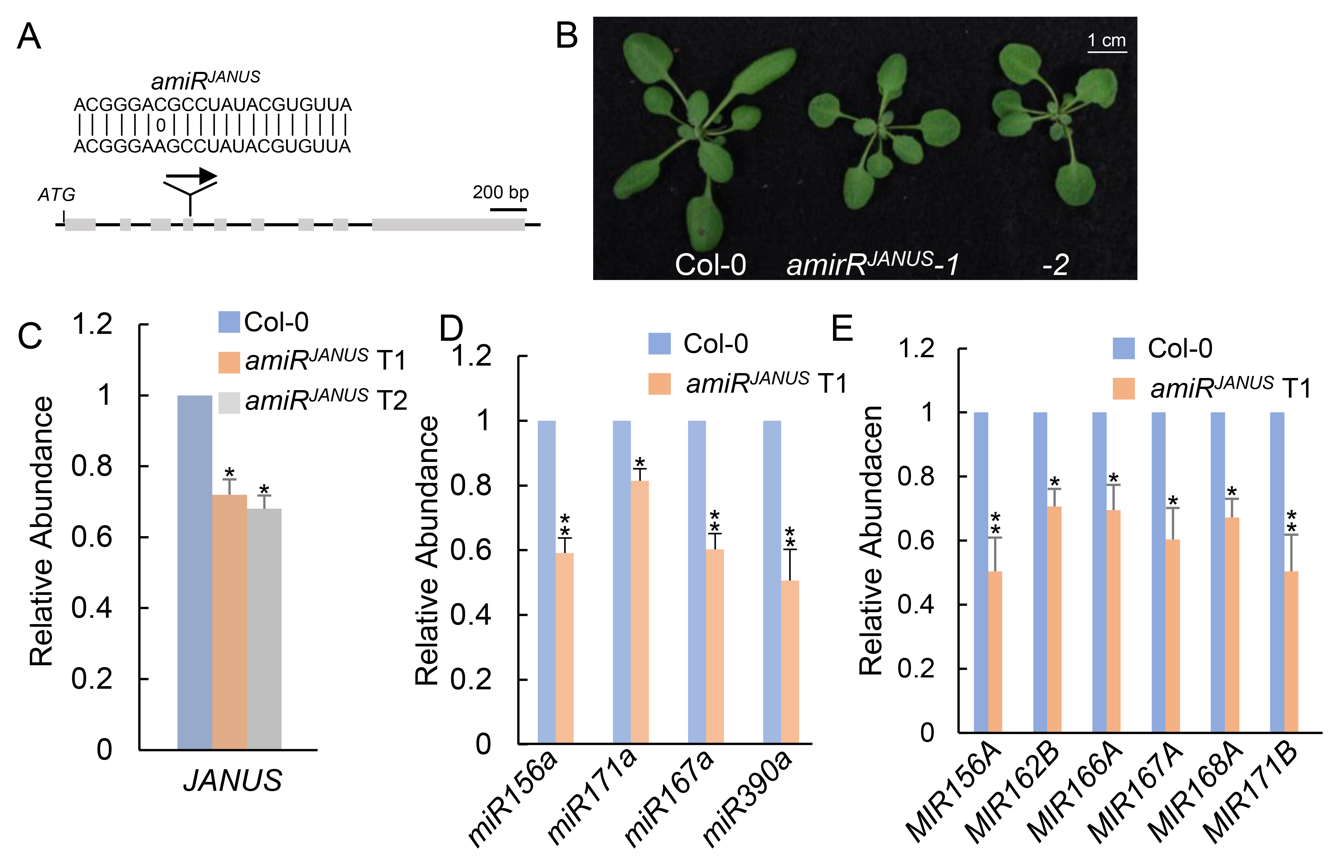
**

**Supplementary Figure S3. Knockdown of *JANUS* by *amiR^JANUS^* reduces the accumulation of pri-miRNAs and miRNAs.** (A) Scheme of an artificial miRNA targeting *JANUS*. Solid line represents Watson–Crick pairing and a “0” indicates a C-A mismatch pair. (B) Twenty-five-day-old Col-0 and *amiR^SEAP1^*. (C) The transcript level of *JANUS* in *amiR^JANUS^* lines detected by RT-qPCR. Janus transcript level was normalized to that of *UBQ5* and compared with Col-0 (value set as 1). Error bars: standard deviations (SD) of three replicates. **p < 0.01, *p < 0.05 (Student’s t test). (D) The accumulation of miRNAs in Col-0 and *amiR^JANUS^* determined by RT-qPCR. miRNA levels in *amiR^JANUS^* were normalized to those of *U6* and compared with Col-0 (set as 1). Error bars: standard deviations (SD) of three replicates. **p < 0.01, *p < 0.05 (Student’s t test). (E) The accumulation of pri-miRNAs in Col-0 and *amiR^JANUS^* determined by RT-qPCR. Pri-miRNA levels in *amiR^JANUS^* were normalized to those of *UBQ5* and compared with Col-0 (set as 1). Error bars: standard deviations (SD) of three replicates. **p < 0.01, *p < 0.05 (Student’s t test).

**
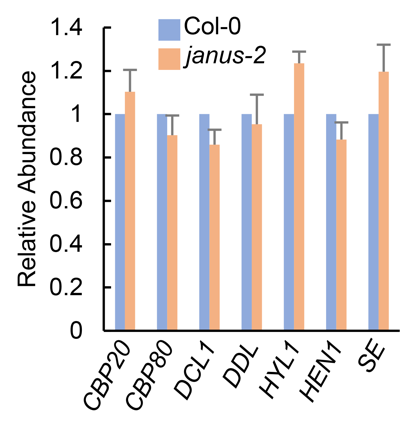
**

**Supplementary Figure S4.** The transcript levels of miRNA biogenesis-related genes in Col-0 and *janus-2*. Transcript levels of examined genes in *janus-2* were determined by RT-qPCR.and normalized to those of *UBQ5* and compared with Col-0 (set as 1). Error bars: standard deviations (SD) of three replicates.

**
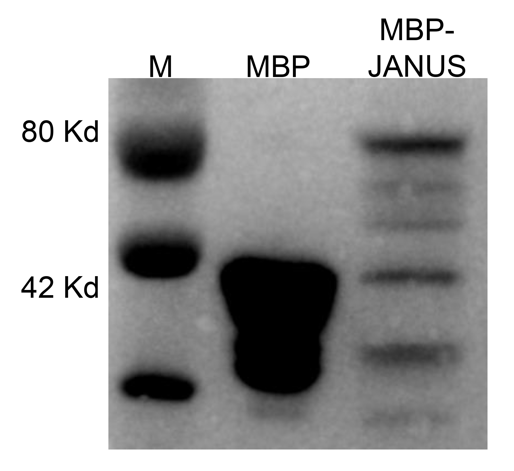
**

**Supplementary Figure S5.** Purified MBP and MBP-JANUS detected by western blot.

**
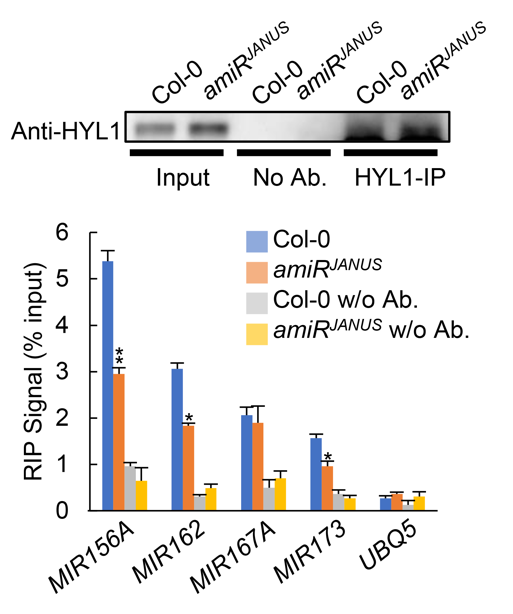
**

**Supplementary Figure S6. The association of HYL1 with pri-miRNAs in *amiR^JANUS^* relative to Col-0 detected by RNA immunoprecipitation (RIP).** IP was performed with the anti-HYL1 antibodies. Detection of HYL1 after IP was shown in upper panel. Ten percent of IPs and 2% input proteins were detected by Western blot. Pri-miRNAs associated with HYL1 were examined by RT-qPCR and normalized to the input. *UBQ5* serves as a negative control. **P < 0.01 (Student’s t test).

**
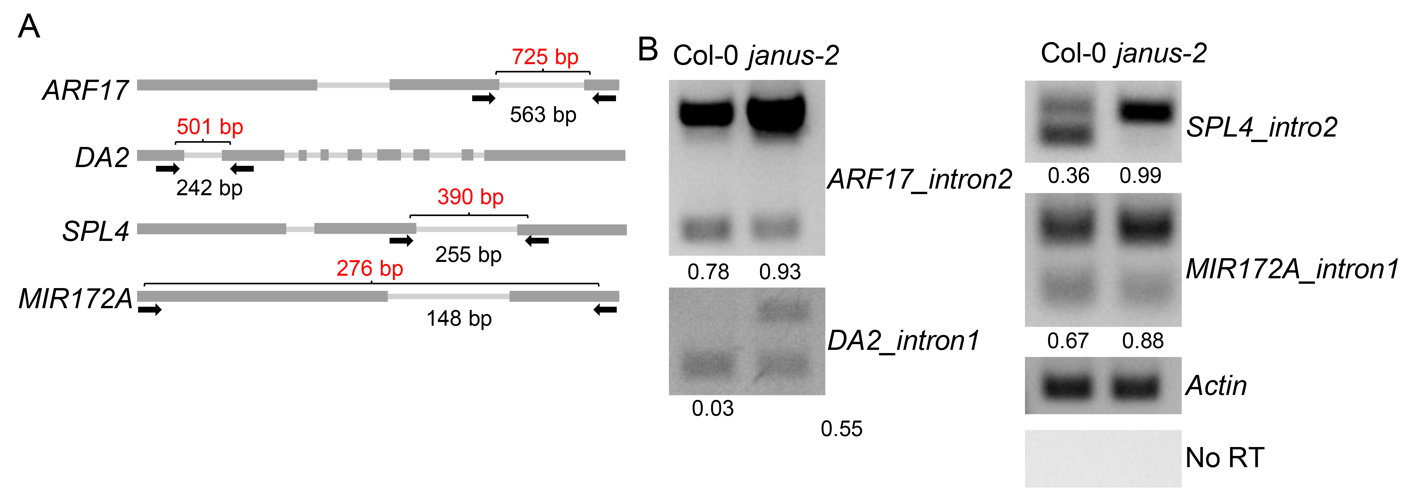
**

**Supplementary Figure S7. The effect of JANUS on splicing of selected genes.** (A) Diagram showing the structures of several differentially spliced transcripts selected for validation. Arrows indicated primer positions used for PCR in (B). The numbers in red indicate the length of PCR products without intron splicing. The numbers in black show the intron length. (B) Intron-retention analysis of selected transcripts by RT-PCR using primers pairs indicated in (A). *ACT2* used as the loading control. The numbers shown below the figures indicate the normalized values relative to Col-0 (set as 1).
