# Supplementary material for: JANUS, a spliceosome-associated protein, promotes miRNA biogenesis in Arabidopsis
Source: Nucleic Acids Res. 2023 Nov 22;52(1):420–30. doi: 10.1093/nar/gkad1105 (PMC10783502; doi:10.1093/nar/gkad1105)
Supplement: gkad1105_Supplemental_Files [file gkad1105_supplemental_files.zip › Supplementary Table 1 primers .docx]

Supplementary Table 1: DNA oligos used in this study.

| **Name** | **Sequence** | **Application** |
| --- | --- | --- |
| **Primers for genotyping** | | |
| CS6053_LP | AGGGTCAAGGTTTCCAATGA | *janus-1* |
| CS16053_RP | TCTTCTCGGCCAACACTCCG |  |
| LB3 | TAGCATCTGAATTTCATAACCAATCTCGATACAC | T-DNA |
| **Primers for constructs** | | |
| JANUSCDS-F | CACCATGACGACTCGAATCGCTCC | JANUSCDS |
| JANUSCDS-R | ACCTTGGTGTGGTGGTGGTG |  |
| JANUS-gF | CACCGCTAAATCTCAACGCTTCTCC | JANUSgenomicfragment |
| JANUSCDS-R | ACCTTGGTGTGGTGGTGGTG |  |
| **Primers for stem-loop PCR** | | |
| miR156dstemloopRT | GTTGGCTCTGGTGCAGGGTCCGAGGTATTCGCACCAGAGCCAACGTTATG | stem-loopPCR |
| miR156dforward | CGGCGGGCTCACTCTCTTTTT | stem-loopPCR |
| miR160astemloopRT | GTTGGCTCTGGTGCAGGGTCCGAGGTATTCGCACCAGAGCCAACTGGCAT | stem-loopPCR |
| miR160aforward | CCTAGCATGCCTGGCTCCCTGT | stem-loopPCR |
| miR167astem-loopRT | GTTGGCTCTGGTGCAGGGTCCGAGGTATTCGCACCAGAGC  CAACTAGATC | stem-loopPCR |
| miR167aForward | GGCGTCTGAAGCTGCCAGCAT | stem-loopPCR |
| miR172a | GTTGGCTCTGGTGCAGGGTCCGAGGTATTGGCACCAGAGCCAACATGCAG |  |
| miR172a | GGCGTCAGAATCTTGATG |  |
| miR173stemloopRT | GTTGGCTCTGGTGCAGGGTCCGAGGTATTCGCACCAGAGC  CAACGTGATT | stem-loopPCR |
| miR173forward | GTTGGCTTCGCTTGCAGAGAG | stem-loopPCR |
| miR399stemloopRT | GTTGGCTCTGGTGCAGGGTCCGAGGTATTCGCACCAGAGC  CAACCAGGGC |  |
| miR399forward | CGGCGGTGCCAAAGGAGATTT |  |
| U6stemloopRT | GTGCAGGGTCCGAGGTTTTGGACCATTTCTCGAT | stem-loopPCR |
| U6forward | GGAACGATACAGAGAAGATTAGCA | stem-loopPCR |
| Universal | GTGCAGGGTCCGAGGT | stem-loopPCR |
| **Primers for RT-PCR** | | |
| JANUSqF | CCTGGTGTTGGAGCTAATCTTCTCG | RT-PCR |
| JANUSqR | CACATCAGGGTCAAGGTTTCCAATG | RT-PCR |
| AFR6_qF | GGGGTCCTTTGGTAGGTCGC | RT-PCR |
| AFR6_qR | CGGCCAGGGGTCATCACCAA | RT-PCR |
| AP2_qF | TCCGTGGAGTAGAAGCGGATATCAA | RT-PCR |
| AP2_qR | TCCCAACGACCACACTTATGCAA | RT-PCR |
| TOE1_qF | CGGGGGAGTTCGAAGTATCGAG | RT-PCR |
| TOE1_qR | ACCGGTCGATAAAGAGATTCCCAAG | RT-PCR |
| TAS2_qF | TTGGGTTTGGGAGTGAGTTTACGAGTTACA | RT-PCR |
| TAS2_qR | GTGAATAGTTTAAGTATCATCATTCGCTTGGA | RT-PCR |
| PHO2_qF | GTGAAGGACCATTTTACGCACC | RT-PCR |
| PHO2_qR | CCATATAAGCCTTGCACGCAG | RT-PCR |
| CBP20qF | ACCGGCCTATTCGTGTGGATTTTG | RT-PCR |
| CBP20qR | TGCCTTTGTGCTTCGAGTTCCTTC | RT-PCR |
| HYL1qF | TTGCCTGGATTCTTCAATCGTAAGG | RT-PCR |
| HYL1qR | TAGGTTCTTGCATAATCCCGTTTCG | RT-PCR |
| SEqF | CCACCGCCTCGTAGGGATTACA | RT-PCR |
| SEqR | CCACCATGGTCATACCCAAATCTTC | RT-PCR |
| DDLqF | ATGAGCCCCCAGAGGCTAGAAAAC | RT-PCR |
| DDLqR | CTGCAAGATGGGTGATCCGTAGGAA | RT-PCR |
| HEN1qF | TTAGGATGACACCCCCTGATGCTG | RT-PCR |
| HEN1qR | AAAAGCCGCCTCCATTCGTTCTTC | RT-PCR |
| DCL1qF | CGTTGTTATGCGTTTCGACCTTGC | RT-PCR |
| DCL1qR | AACGCTGCGTGAGATACATTTCCTC | RT-PCR |
| MIR156aqF | AAGGGGGTCTTCTATCATCAGGA | RT-PCR/RIP |
| MIR156aqR | TGATTGGAATATGCCCTAAAGAGTG | RT-PCR/RIP |
| MIR158aqF | GTGATGACGCCATTGCTCTTT | RT-PCR/RIP |
| MIR158aqR | TGTGACTTTAGATGCCCTTGTTCA | RT-PCR/RIP |
| MIR166aqF | GACTCTGGCTCGCTCTATTCA | RT-PCR/RIP |
| MIR166aqR | TGGTCCGAAGACGCTAAAAC | RT-PCR/RIP |
| MIR167aqF | TGTTGTGTTTCATGACGATGG | RT-PCR/RIP |
| MIR167aqR | AGCTCACAAAATCAGACTGAAGA | RT-PCR/RIP |
| MIR168aqF | AGTAGAGTCTCACCATCGGGCT | RT-PCR/RIP |
| MIR168aqR | TTACACCTCGAGGATCCGATT | RT-PCR/RIP |
| MIR171aqF | tgctttggtagtagatgaggtt | RT-PCR/RIP |
| MIR171aqR | CGTGTGTGGTCAGGTAAGAT | RT-PCR/RIP |
| MIR319bqF | AGCTTTCTTCGGTCCACTCATGG | RT-PCR/RIP |
| MIR319bqR | GAGCTCCCTTCAGTCCAAGCATA | RT-PCR/RIP |
| ACT2qF | GAAATCACAGCACTTGCACC | RT-PCR |
| ACT2qR | AAGCCTTTGATCTTGAGAGC | RT-PCR |
| UBQ5-N | GGTGCTAAGAAGAGGAAGAAT | RT-PCR |
| UBQ5-C | CTCCTTCTTTCTGGTAAACGT | RT-PCR |
| **Primers for ChIP PCR** | | |
| PolII-C1-F | AGTTCAATGGAGAGATGTCGAAATATG | ChIP-PCR |
| PolII-C1-R | AAGAGGAAAAGAAAGAGATGGAGAGA | ChIP-PCR |
| cMIR156a-F | GAATGGTCTTTTGATGATGGAAGG | ChIP-PCR |
| cMIR156a-R | TCCGCCAACAAAGGAAAGAGA | ChIP-PCR |
| cMIR164A-F | TGCACGTACTTAACTTCTCCA | ChIP-PCR |
| cMIR164A-R | ACACTTACATATATGAGAACT | ChIP-PCR |
| cMIR166a-F | TGGCTCTCTCCACTACTCAA | ChIP-PCR |
| cMIR166a-R | GACAACAGTCCCCTCAAAA | ChIP-PCR |
| cMIR167a-F | CGACCCTTAAACTCTCCATAA | ChIP-PCR |
| cMIR167a-R | ACTTCACCGTAGCAGATCAA | ChIP-PCR |
| cMIR168a-F | AACACATTCACATACATTACGTTGG | ChIP-PCR |
| cMIR168a-R | TATTTGGAAAAGATTAGAACAGCG | ChIP-PCR |
| **Primers for *in vitro* RNA binding** | | |
| T7miR162b-p3 | TAATACGACTCACTATAGGGAAAGAGTGAAGTCGCTGGAG | pri-miR162bProbe |
| miR162b-p4 | CATGAAGAGCAAGCAGCGCTGGATGC | pri-miR162bProbe |
| T7-premiR162bF | TAATACGACTCACTATAGGAGGCAGCGGTTCATCGATC | pre-miR162bProbe |
| premiR162bR | CTGGATGCAGAGGTTTATCGATC | pre-miR162bProbe |
| T7-premiR172bF | TAATACGACTCACTATAGGGGTCGTTGTTTGTAGGCGCAG | pre-miR172bProbe |
| premiR172bR | TTTGTAGCCGTCGATTGTTG | pre-miR172bProbe |
| UBQ5T7F | TAATACGACTCACTATAGGGATGCAGATCTTCGTGAAAACC | ssRNAprobe |
| UBQR2 | GGATTCCTTCCTTGTCTTGGA | ssRNAprobe |
| RNA/RNAblunt1 | TAATACGACTCACTATAGGGCAACAACTGTCTCTTGGATCG  TATATTGCCATTTATGTGTTGAGCCC | dsRNAprobe |
| RNA/RNAblunt2 | TAATACGACTCACTATAGGGCTCAACACATAAATGGCAAT  ATACGATCCAAGAGACAGTTGTTGCCC | dsRNAprobe |
| **Primers for intron splicing** | | |
| iDA2-F | CTGTATTATCTTTGCTTCTCTCTTCTCT | pre-mRNAsplicing |
| iDA2-R | CTCACATTAACACCTAAAATTCAATTTGTT | pre-mRNAsplicing |
| iSPL4_F | TGCAGAATCAAGAAAGATCAA | pre-mRNAsplicing |
| iSPL4_R | TTAGTAGTTTCCCTGTGCTTTTG | pre-mRNAsplicing |
| iARF17_F | GTCTGGCTTGTGTGAAGAGGA | pre-mRNAsplicing |
| iARF17_R | TTCCAGCAAAGATCCACCACT | pre-mRNAsplicing |
| inpri-miR172a-F | ATGCTGCATCGGCAATCAACGACT | pri-miRNAsplicing |
| inpri-miR172a-R | GTGGATCTATTAATGTCTTGATAAAGACTGCC | pri-miRNAsplicing |
